# A Comparison of Hospitalized Patients With Heart Failure and Cancer Referred to Palliative Care

**DOI:** 10.1001/jamanetworkopen.2020.0020

**Published:** 2020-02-26

**Authors:** Albert Y Liu, David L. O’Riordan, Angela K. Marks, Kara E. Bischoff, Steven Z. Pantilat

**Affiliations:** 1 Department of Medicine, University of California, San Francisco; 2 Division of Palliative Medicine, University of California, San Francisco

## Abstract

**Importance:**

Growing evidence shows that palliative care (PC) improves treatment outcomes in patients with heart failure (HF), but few large-scale studies have prospectively evaluated the processes and outcomes associated with PC consultation for such patients in the real world.

**Objective:**

To characterize processes and outcomes of PC consultations for hospitalized patients with HF compared with patients with cancer.

**Design, Setting, and Participants:**

This cohort study of inpatient encounters at community and academic hospitals in the Palliative Care Quality Network enrolled participants between 2013 and 2017. Of a total of 135 197 patients, 57 272 adults with a primary diagnosis of HF or cancer receiving PC consultation were enrolled. Data analysis was performed from April 2018 to December 2019.

**Exposures:**

Primary diagnosis of HF or cancer.

**Main Outcomes and Measures:**

Symptom improvement and changes in care planning documentation after PC consultation.

**Results:**

At the time of consultation, patients with HF were older (mean age, 75.3 years [95% CI, 75.0-75.5 years] vs 65.2 years [95% CI, 65.0-65.3 years]; *P* < .001), had lower Palliative Performance Scale scores (mean, 35.6% [95% CI, 35.3%-35.9%] vs 42.4% [95% CI, 42.2%-42.6%]; *P* < .001), and were more likely to be in a critical care unit (5808 of 16 741 patients [35.3%] vs 4985 of 40 531 patients [12.5%]; *P* < .001) or a telemetry or step-down unit (5802 of 16 741 patients [35.2%] vs 7651 of 40 531 patients [19.2%]; *P* < .001) compared with patients with cancer. Patients with HF were less likely than patients with cancer to be referred to PC within 24 hours of admission (6773 of 16 741 patients [41.2%] vs 19 348 of 40 531 patients [49.0%]; *P* < .001) and had longer hospitalizations before receiving PC consultation requests (mean, 4.6 days [95% CI, 4.4-4.8 days] vs 3.9 days [95% CI, 3.8-4.0 days]; *P* < .001). Patients with HF were referred less frequently for symptoms other than pain (1686 of 16 488 patients [10.2%] vs 8587 of 39 609 patients [21.7%]; *P* < .001), but were equally likely to report improvements in anxiety (odds ratio, 0.85; 95% CI, 0.71-1.02; *P* = .08) and more likely to report improvements in dyspnea (odds ratio, 2.17; 95% CI, 1.83-2.57; *P* < .001) compared with patients with cancer. Patients with HF were less likely than those with cancer to be discharged alive (odds ratio, 0.78; 95% CI, 0.64-0.96; *P* = .02) or to be referred to hospice (odds ratio, 0.50; 95% CI, 0.47-0.53; *P* < .001).

**Conclusions and Relevance:**

These findings suggest that PC referral comes late for patients with HF and is used primarily to discuss care planning. Practitioners caring for patients with HF should consider involving PC experts earlier for symptom management.

## Key Points

**Question:**

How do hospitalized patients with heart failure who are referred to palliative care differ from patients with cancer in their characteristics, reasons for referral, and outcomes of referral?

**Findings:**

In this cohort study of 57 272 adults, patients with heart failure were referred for palliative care later in their disease course than patients with cancer. Fewer patients with heart failure were referred for symptom management but they had similar rates of symptom improvement after referral.

**Meaning:**

Hospitalized patients with heart failure are being referred later to palliative care, suggesting that there are opportunities to integrate palliative care earlier in the disease trajectory for symptom management.

## Teaser

This cohort study evaluates the processes and outcomes of palliative care consultations for hospitalized patients with heart failure compared with patients with cancer at community and academic hospitals included in the Palliative Care Quality Network.

## Introduction

Despite significant advances in disease-modifying therapies over the last few decades, the morbidity and mortality associated with heart failure (HF) remain high.^1,2,3,4,5,6^ Heart failure is the leading cause of hospitalizations among patients older than 65 years. Furthermore, more than 20% of patients die within a year of initial hospitalization.^1,2,6^ Patients with HF have a myriad of symptoms, including dyspnea, fatigue, pain, and depression.^4,5,7,8,9,10^ As the disease progresses, many patients face difficult decisions regarding treatments, such as left ventricular assist devices, cardiac transplantation, and repeated hospitalization.^11,12^

Palliative care (PC) is a medical specialty focused on improving quality of care by managing symptoms, elucidating and clarifying goals of care, and providing psychological, emotional, social, and spiritual support to people with serious illness and their families.^13,14^ Consultation for PC provided alongside optimal HF management has been shown to reduce rates of depression, decrease symptom burden, and enhance quality of life for patients with HF in both outpatient and hospitalized inpatient settings.^15,16,17^ Despite consensus guidelines from multiple societies recommending integration of PC early in the HF disease trajectory,^11,18,19^ PC for HF remains underutilized. Patients with HF have poor functional status at the time of referral.^20,21^ Compared with patients with cancer, patients dying of refractory HF in the United States are also less likely to be receive hospice services and are more likely to die in the hospital.^22,23,24,25^ Given the need for improved and earlier integration of PC in HF management, the purpose of this study is to compare patient characteristics, processes of care, and treatment outcomes of hospitalized patients with HF and cancer using the Palliative Care Quality Network (PCQN) data set.

## Methods

The study was reviewed and approved by the University of California, San Francisco, institutional review board. The need for informed consent for participants was waived because PCQN data are deidentified and aggregated in order to evaluate quality of care provided to patients by PC teams.

### Palliative Care Quality Network

The PCQN is a nationwide collaborative of interdisciplinary PC teams collecting standardized data on processes of care and patient-level outcomes to determine best practices and promote quality improvement.^26^ As of December 2017, there were 88 teams from 17 states collecting and submitting data. Hospitals in the PCQN varied in size (mean, 379 beds; range, 48-1126 beds) and type (not-for-profit, 66%; teaching, 19%; public, 9%; for-profit, 1%; and other, 4%).

### Data Set

The PCQN data set includes patient demographic characteristics (eg, age, sex, primary diagnosis, and functional status measured by the Palliative Performance Scale [PPS])^27^ and information about the context of the consultation (eg, date of PC consultation request and reasons and primary diagnosis prompting the PC consultation). The PPS is a tool developed by the Victoria Hospice Society that measures functional status by evaluating patients’ ambulation, activity level, self-care, intake, and consciousness level. The PPS results in a score from 100% (normal) to 0% (dead), with higher scores indicating better functional status. For patients able to report on a 4-point ordinal scale (none, mild, moderate, or severe), symptom severity scores for pain, anxiety, dyspnea, and nausea are assessed daily. The PCQN data set also documents information about care planning, family meetings, and disposition.

### Procedure

The data for this study were extracted on March 6, 2018, and include data from 135 197 patients who received a referral for a PC consultation from 1 of 88 PCQN member teams between January 1, 2013, and December 31, 2017. Data analysis was performed from April 2018 to December 2019.

### Statistical Analysis

Descriptive statistics (frequency, mean, SD, and range) were calculated to examine the distribution of measures, including PPS score, patient-reported symptom severity, and improvement in symptoms at follow-up PC assessment. For the symptom severity analyses, only patients who were able to report their symptoms were included. Bivariate associations between independent variables and dependent variables, such as patient characteristics, processes of care, and outcomes, were done using χ^2^ analysis and analyses of variance. Logistic regression analyses were undertaken to examine characteristics and processes of care for patients with HF. To account for multiple comparisons, a more conservative 2-sided α < .01 was used to determine statistical significance. We included covariates that were statistically significant in bivariate analyses, and PC teams were included as a random effect to account for patient clustering. There was no adjustment or imputation for missing data. Analyses were performed only for patients for whom data were available for each specific data element, resulting in different *n* values for each analysis. SPSS statistical software version 23 for Mac (IBM) was used to conduct all analyses.

## Results

Of the 135 167 total PCQN patient encounters, we analyzed 57 272 patients: 40 531 (30.0%) patients with a primary diagnosis of cancer and 16 741 (12.4%) with a primary diagnosis of HF, with considerable variability among PC teams (range, 0%-59.0%). Patients with HF were older (mean age, 75.3 years [95% CI, 75.0-75.5 years] vs 65.2 years [95% CI, 65.0-65.3 years]; *P* < .001) and had lower PPS scores (mean, 35.6% [95% CI, 35.3%-35.9%] vs 42.4% [95% CI, 42.2%-42.6%]; *P* < .001) than patients with cancer at the time of referral (Table 1). Patients with HF were more likely than those with cancer to be referred for PC consultation from a critical care unit (5808 of 16 741 patients [35.3%] vs 4985 of 40 531 patients [12.5%]; *P* < .001) or telemetry or step-down unit (5802 of 16 741 patients [35.2%] vs 7651 of 40 531 patients [19.2%]; *P* < .001). Patients with HF were referred more often for care planning (13 564 of 16 741 patients [82.3%] vs 26 885 of 40 531 patients [67.9%]; *P* < .001) and less often for management of pain (1128 of 16 741 patients [6.8%] vs 13 839 of 40 531 patients [34.6%]; *P* < .001) and other symptoms (1686 of 16 488 patients [10.2%] vs 8587 of 39 609 patients [21.7%]; *P* < .001) compared with patients with cancer. Patients with HF were less likely than those with cancer to be referred within 24 hours of hospital admission (6773 of 16 741 patients [41.2%] vs 19 348 of 40 531 patients [49.0%]; *P* < .001) and had longer hospitalizations before receiving PC consultation requests (mean 4.6 days [95% CI, 4.4-4.8 days] vs 3.9 days [95% CI, 3.8-4.0 days]; *P* < .001). They were followed up for a similar length of time after the PC consultation (5.5 days [95% CI, 5.3-5.7 days] vs 5.5 days [95% CI, 5.4-5.6 days]; *P* = .60). At the time of consultation, 11.6% (1812 of 15 612) of patients with HF had physician orders for life-sustaining treatment form (or their equivalent) completed, 24.3% (3903 of 16 070) had advanced directives completed, and 38.4% (6196 of 16 141) had a code status of do not resuscitate or do not intubate. Patients with HF, compared with patients with cancer, had lower rates of moderate-to-severe pain (1102 of 8176 patients [13.5%] vs 10 850 of 25 939 patients [41.8%]; *P* < .001), anxiety (714 of 7730 patients [9.2%] vs 3585 of 23 761 patients [15.1%]; *P* < .001), and nausea (159 of 8038 patients [2.0%] vs 2302 of 25 321 patients [9.1%]; *P* < .001), but had significantly higher rates of moderate-to-severe dyspnea (1232 of 8132 patients [15.2%] vs 2544 of 25 399 patients [10.0%]; *P* < .001).

**Table 1.** Patient Characteristics at Time of Referral to Palliative Care Consultation

Characteristic	Patients, No. (%)		*P* Value
	Cancer	Heart Failure	
Total^a^	40 531 (30.0)	16 741 (12.4)	
Age, mean (95% CI), y	65.2 (65.0-65.3)	75.3 (75.0-75.5)	<.001
Male, No./total No. (%)	19 878/40 522 (49.1)	9156/16 736 (54.7)	<.001
Palliative Performance Scale score, mean (95% CI), %	42.4 (42.2-42.6)	35.6 (35.3-35.9)	<.001
Referral location			
Total patients, No.	39 846	16 476	<.001
Medical or surgical unit	21 854 (54.8)	3368 (20.4)	
Critical care unit	4985 (12.5)	5808 (35.3)	
Telemetry or step-down unit	7651 (19.2)	5802 (35.2)	
Other	5356 (13.4)	1498 (9.1)	
Reason for referral^b^			
Total patients, No.	39 609	16 488	<.001
Goals of care or advanced care planning	26 885 (67.9)	13 564 (82.3)	
Pain management	13 839 (34.6)	1128 (6.8)	
Other symptom management	8587 (21.7)	1686 (10.2)	
Hospice referral or discussion	6679 (16.9)	2489 (15.1)	
Transfer to comfort care	2236 (5.6)	1328 (8.1)	
Withdrawal of interventions	611 (1.5)	612 (3.7)	
Assess for transfer to comfort care	843 (2.1)	388 (2.4)	.10
Support for patient or family, No./total No. (%)	8291/39 615 (20.9)	4423/16 490 (26.8)	<.001
Physician orders for life-sustaining treatment at time of consultation, No./total No. (%)	3317/38 314 (8.7)	1812/15 612 (11.6)	<.001
Advance directive at time of consultation, No./total No. (%)	8180/38 658 (21.2)	3903/16 070 (24.3)	<.001
Code status at time of consultation			
Total patients, No.	38 731	16 141	<.001
Full	23 926 (61.8)	9017 (55.9)	
Partial	1598 (4.1)	928 (5.7)	
Do not resuscitate or do not intubate	13 207 (34.1)	6196 (38.4)	
Time between admission and palliative care consultation request, mean (95% CI), d	3.9 (3.8-4.0)	4.6 (4.4-4.8)	<.001
Patients receiving a request for palliative care referral within 24 h of admission, No./total No. (%)	19 348/39 467 (49.0)	6773/16 433 (41.2)	<.001
Moderate or severe symptoms at time of consultation, patients, No./total No. (%)			
Pain	10 850/25 939 (41.8)	1102/8176 (13.5)	<.001
Anxiety	3585/23 761 (15.1)	714/7730 (9.2)	<.001
Nausea	2302/25 321 (9.1)	159/8038 (2.0)	<.001
Dyspnea	2544/25 399 (10.0)	1232/8132 (15.2)	<.001

^a^ Percentages reflect frequency of all 135 197 patients.

^b^ Percentages do not add up to 100% because multiple reasons for referral can be selected.

After adjusting for all statistically significant characteristics in the univariate analyses and accounting for the patient clustering among PCQN teams, multivariate logistic regression showed that compared with patients with cancer, patients with HF had similar rates of physician orders for life-sustaining treatment form completion (odds ratio [OR], 1.09; 95% CI, 0.91-1.32) and advanced directive completion (OR, 1.02; 95% CI, 0.78-1.33) after PC referral (Table 2). They were less likely to experience improvements in pain (OR, 0.41; 95% CI, 0.35-0.48) or nausea (OR, 0.39; 95% CI, 0.30-0.51; *P* < .001), but were equally likely to have improvements in anxiety (OR, 0.85; 95% CI, 0.71-1.02; *P* = .08) and more likely to have improvements in dyspnea (OR, 2.17; 95% CI, 1.83-2.57; *P* < .001) compared with patients with cancer. Patients were HF were less likely than patients with cancer to be discharged alive (OR, 0.78; 95% CI, 0.64-0.96; *P* = .02). Of patients discharged alive, 31.5% of patients with HF were referred to hospice. After adjusting for age, sex, functional status, and clustering within PC teams, we found that patients with HF discharged alive were 50% less likely to be referred to hospice than patients with cancer (OR, 0.50; 95% CI, 0.47-0.53; *P* < .001).

**Table 2.** Multivariate Analysis of Processes of Care for Patients With Heart Failure Compared With Patients With Cancer^a^

Variable	Odds Ratio (95% CI)	*P* Value
Family meetings	0.89 (0.85-0.95)	<.001
Code status at discharge		
Partial	1.39 (0.92-2.08)	.11
Do not resuscitate or do not intubate	0.87 (0.74-1.03)	.10
Physician orders for life-sustaining treatment completed at discharge	1.09 (0.91-1.32)	.36
Advanced directives completed at discharge	1.02 (0.78-1.33)	.36
Any improvement in symptoms from first to second assessment		
Pain	0.41 (0.35-0.48)	<.001
Anxiety	0.85 (0.71-1.02)	.08
Nausea	0.39 (0.30-0.51)	<.001
Dyspnea	2.17 (1.83-2.57)	<.001
Discharged alive	0.78 (0.64-0.96)	.02

^a^ Age, sex, and Palliative Performance Scale score were included as covariates, and palliative care teams were included as random effects.

## Discussion

Heart failure is the most common reason for hospital admission among older people.^1,6^ However, among PCQN hospitals, only 12.4% of referrals for specialty PC were for patients with HF, which is less than half the number of referrals received for patients with cancer. Compared with patients with cancer, patients with HF were referred later during their hospitalizations and were older, had lower functional status, and were more likely to be in higher acuity units at the time of referral. Despite these characteristics, they were also less likely to be referred to hospice at discharge compared with patients with cancer. These findings are consistent with existing literature describing delayed referral to PC and hospice services for people with HF.^8,9,10,21,22,23,24,25^ Encouragingly, the hospice referral rate after PC consultation in our study was much higher than previously described hospice referral rates for hospitalized patients with HF; this may be explained by the fact that all the patients were seen by specialty PC teams with expertise in discussing hospice referral.^28^

Patients with HF have a high symptom burden, and consensus guidelines describe symptom control as an integral part of HF management.^1,11,12,18^ Yet in our study, only a small fraction of patients was referred for management of symptoms other than pain. It is unclear why this gap exists, but it may be associated with perceptions among health care practitioners that PC services are helpful only for patients with refractory HF or those who are dying.^29^ Because many traditional interventions for HF, such as diuresis and inotropy, often improve symptoms, practitioners managing patients with HF may not think that PC consultation is necessary. Another possibility is that practitioners are more attuned to pain than other symptoms,^30^ and pain is less prevalent among patients with HF. What is notable is that patients with HF who were referred to PC had similar improvements in anxiety and were much more likely to have improvements in dyspnea compared with patients with cancer. Because of the lack of data on treatments provided to patients, it is difficult to know which specific interventions led to this improvement, but these findings support the idea that PC consultation adds value for symptom management beyond typical disease-modifying therapies.^16^

### Limitations

There are limitations to the conclusions that can be drawn from this study. It is a descriptive, prospective study using data collected by PC teams; thus, we are unable to compare the care of patients with HF who were not referred for PC consultation. Because patients with HF were more ill and more often referred from the intensive care unit than patients with cancer, we may be underreporting their symptom burden. Symptom improvement after initial consultation may have resulted from traditional interventions, such as diuresis, and may not fully reflect the outcomes of interventions by PC teams. However, the fact that patients with HF were in the hospital for more than 4 days before PC consultation suggests that there was time for diuresis and that the improvement after consultation likely represents the contributions of the PC team, including the use of opioids. Previous studies^31,32,33^ using PCQN data have described additional limitations, most notably that these data were collected by PC teams over the course of usual patient care rather than by a research assistant following a specific protocol. The PCQN data set includes only those data elements determined to be useful for real-time clinical care and informing quality improvement initiatives. Unfortunately, this limits our ability to collect more granular data, such as factors leading to delayed referral or symptom improvement for patients with HF. In addition, given the large sample size, statistically significant results may not necessarily be clinically meaningful. We try to remain cognizant of this issue as we interpret and report our findings and acknowledge that care needs to be taken in the interpretation of the results.

## Conclusions

Systematic, interdisciplinary PC intervention for patients with advanced HF improves quality of life and reduces distress associated with symptoms.^15^ However, what remains unclear is at what stage in the disease trajectory these interdisciplinary teams should be used. Current society guidelines^11,18,19^ already describe a need for early integration of PC in HF, but our study shows that this may not be happening in practice. Hospitalization is a sentinel moment in the HF disease course and may be an opportunity to get PC experts involved, especially given the high mortality rates after index hospitalization. Future studies need to explore the benefits of earlier PC integration on HF treatment outcomes, before the disease is at the end stage. In addition, more work needs to be done to tease out which specific PC interventions lead to meaningful improvements in patient outcomes. Routine symptom monitoring has been shown to improve survival for patients with metastatic cancer undergoing chemotherapy^34^ and may be of benefit in patients with HF. For now, cardiologists, primary care physicians, and hospitalists taking care of patients with HF should consider involving PC specialists early not only for care planning but also for assistance with symptom management.
